# The role of the behavioral immune system in the expression of short and long-term orientation in young Chilean men during the COVID-19 pandemic

**DOI:** 10.1186/s12889-025-21755-y

**Published:** 2025-02-07

**Authors:** Oriana Figueroa, Pablo Polo, Daniel Torrico-Bazoberry, Gabriela Fajardo, Carlos Rodríguez-Sickert, Nohelia Valenzuela, Abigail Arenas, Paula Pavez, Montserrat Belinchon, Gabriela Valdebenito, José Antonio Muñoz-Reyes

**Affiliations:** 1 Facultad de Salud y Ciencias Sociales, Universidad de Las Américas, Santiago, Chile; 2 Laboratorio de Comportamiento Animal y Humano, Centro de Investigación en Complejidad Social, Facultad de Gobierno, Universidad del Desarrollo, Av. Las Condes 12461, Edificio 3, Piso 3, Santiago, Chile; 3 Facultad de Educación, Universidad San Sebastián, Santiago, Chile; 4 Facultad de Administración y Economía, Universidad de Santiago de Chile, Santiago, Chile

**Keywords:** Sociosexuality, COVID-19, Behavioral immune system, Pandemic, Men

## Abstract

**Introduction:**

The COVID-19 pandemic drastically changed people's lives. It had consequences at the individual and social level. The behavioral immune system predicts that when faced with the risk of contagion from pathogens, people tend to reduce their sociality, especially sociosexuality. We examine this prediction by evaluating decreases in the pandemic of the different dimensions of sociosexuality of young men (i.e., short and long-term mating orientation, sexual desire, and sociosexual behavior) and considering their relationship status (single or paired).

**Methods and materials:**

We compared data from two cross-sectional studies carried out in the laboratory with convenience sampling methods in the pre-pandemic period (in the years 2016 and 2018 with a sample size of N = 463) and pandemic (face-to-face panstudy N = 234,data online, N = 182), considering possible differences between samples of the same period. We reached an N = 879 young men who answered a sociodemographic questioarticipants answered a sociodemographic questionnaire that inclnnaire and the Multidimensional Sociosexual Orientation Inventory. To test our predictions, we fitted general linear models.

**Results:**

Results point to a significant decrease in long-term mating orientation in paired and single men, but only when compared with the pandemic face-to-face study. In addition, no differences were found for short-term mating orientation. For sociosexual desire (i.e., sexual fantasies), we found a reduction in single pre-pandemic individuals to be compared with the sample of pandemic online study. Finally, we found a decrease in sociosexual behavior (i.e., number of partners in the last year) between pre-pandemic samples and the pandemic itself, regardless of their relationship status and samples inside of each period. In addition, we carry out analyses with a reduced sample to re-test our predictions based on the perception of contagion risk (measured by a survey from OMS).

**Conclusion:**

We find changes are maintained at the level of sociosexual desire and sexual behavior but mainly in those individuals with a greater perception of the risk of contagion. The hypotheses derived from the behavioral immune system regarding the decrease in sociosexuality in a context of risk of contagion by pathogens, as was the case in the initial period of COVID-19, are corroborated only in terms of desire and behavior, which suggests a certain stability in attitudes i.e., the menace of contagion modifies proximal aspects of sociosexuality, such as fantasies and behavior, but has no apparent effect on the calibration of the predisposition of individuals to varying levels of commitment in sexual relationships. These findings will allow us to understand better how the dimensions of sociosexuality (i.e., fantasies, attitudes and behavior) are affected when there are contexts of high risk of contagion, such as COVID-19. These findings will allow us to understand better how sociosexuality is affected when there are contexts of high risk of contagion, such as COVID-19.

**Supplementary Information:**

The online version contains supplementary material available at 10.1186/s12889-025-21755-y.

## Introduction

The COVID-19 pandemic has resulted in millions of deaths worldwide and has had widespread effects at biological, psychological, and social levels [5, 28, 49]. This disease, caused by the coronavirus (SARS-CoV-2), is classified as severe pneumonia at a biological level, with a moderate mortality rate and high contagion among individuals [49]. Psychologically and socially, the pandemic has significantly impacted people's lives and routines globally. Lockdowns and social isolation have had adverse effects on mental health, leading to increased rates of depression and anxiety [28], as well as reports of symptoms of post-traumatic stress, fear of infection, boredom, anger, and frustration [5]. Despite the negative consequences of forced confinement and social isolation, the reduction of social contact and interactions during high-risk infection contexts is considered to be an adaptive behavioral response aimed at preventing the spread of infectious diseases [36, 37]. This partially explains why many people choose to isolate themselves.

Schaller [36] and Schaller and Duncan [37] proposed the presence of a behavioral mechanism involving several psychological processes that function to identify and avoid contagious diseases. The so-called behavioral immune system aims to reduce the high physiological costs involved in the physiological immune system response, the possibility of sequelae derived from sickness, and, ultimately, the risk of death. Therefore, the behavioral immune system functions as a proactive behavioral response to reduce exposition to pathogens [37, 39]. There is evidence in several animal species of a behavioral immune system, primarily focused on detecting signals of sick conspecifics and avoiding contact with them [4, 8, 23, 46]. The mental functioning of the behavioral immune system is articulated from the error management theory (EMT) [17], but see [18] for a general application of this theory beyond mating). The EMT states for the behavioral immune system that when the costs of being wrong in a decision—for example, by interacting with someone sick because we have dismissed their signs of infection- outweigh the benefits of interacting with that person, it is always better to take any sign of infection as positive, no matter how superficial or confounding it is (e.g., cough, hoarseness, sneezing, etc.). Following this behavioral rule, the behavioral immune system establishes a series of predictions that involve behavioral changes in response to the perception of a threat of infection by pathogens in conspecifics [1]. These predictions involve prophylactic behaviors that decrease sociability at different levels, including social interaction and gregariousness, normative behavior, and sexual behavior [36]. In this sense, the presence of infection risk is expected to reduce social interactions, especially to avoid contact with unfamiliar and outgroup people, which can produce serious problems such as an increase in xenophobia or racism. In addition, it predicts a reduction in the mental predisposition to new experiences, leading to an increase in conservatism and higher conformity to social norms. Finally, regarding sociosexuality, it is expected that promiscuity and attitudes towards short-term mating would decrease since sexual contact with others entails a high risk of infection. The COVID-19 pandemic gave the exceptional possibility to test the main predictions of the behavioral immune system, with the constriction of sociosexuality being a crucial part of this model.

Sociosexuality refers to an individual's inclination for sexual relationships with varying levels of commitment [43]. This concept has garnered attention in studies of human mating as it can be used to gauge mating efforts toward short-term or long-term relationships [15, 21, 43]. Sociosexuality entails attitudinal (i.e., short and long-term mating orientation), behavioral (i.e., number of previous sexual partners), and desire (i.e., subjective sexual arousal and sexual fantasies of uncommitted partners) components [21, 34]. According to this conceptualization, sociosexual attitudes involve two independent dimensions reflecting motivations and attitudes toward get involved in uncommitted relationships with a diversity of mating partners (short-term mating orientation), on the one hand, and motivations and attitudes toward the search for a committed long-lasting relationship, on the other (long-term mating orientation) [21]. This distinction between short and long-term mating orientation reflects more accurately the pluralism described in human mating strategies [15]. According to the behavioral immune system, the likelihood of infection should diminish attitudes toward short-term mating and we don´t expected changes in long-term mating orientation [36]. Following this prediction, previous studies indicated that there was a negative association between short-term mating orientation and the perception of the risk of infection in humans [9]. Additionally, a cross-cultural study revealed that in regions with a historically high prevalence of diseases, women reported lower levels of short-term mating orientation, which supports the proposal of the behavioral immune system [38]. Men exhibited a similar trend to women, but the association was weaker and not statistically significant, raising doubts about this relationship in men [38]. Further evidence from experimental studies that manipulated the disease thread showed that both men and women scored less in short-term mating orientation compared to a control condition or non-disease condition [30, 33]. However, since the prediction from the behavioral immune system is related to the decrease in promiscuity, it is not clear how the likelihood of infection should affect long-term mating orientation.

During the pandemic outbreak, a decrease in sexual activity has been reported in various studies involving both single individuals and romantic couples [14, 19, 20, 25, 45, 48]. The decrease in sexual activity among single individuals can be attributed to social isolation, which limits sexual relationships. In fact, there is some evidence suggesting that individuals decreased the number of sexual partners during the pandemic [26]. Moreover, it has been observed that men masturbated more often during physical distancing measures and lockdown, suggesting a decrease in sociosexual behavior (number of sexual partners) but not in sexual or sociosexual desires, assuming that increased rates of masturbation are a form of replacement behavior [19, 31]. According to the behavioral immune system proposal, the reported decrease in sexual activity and number of sexual partners during the pandemic should be explained by a decrease in short-term mating orientation and sociosexual desire [36]. However, to our knowledge, there are no studies that have investigated the effect of the pandemic on sociosexual attitudes and desires since this information cannot be measured with retrospective methods, and consequently, it is still unclear whether the changes in patterns of sociosexual behavior are due to a direct negative effect of the behavioral immune system on sociosexual attitudes and desires, or if they are a result of social isolation limiting sociosexual behavior. To address this question, measures of sociosexuality taken before and during COVID-19 are necessary to determine if the behavioral immune system affects short-term mating orientation and the motivation to seek new sexual partners.

The aim of this study is to test the prediction derived from the behavioral immune system regarding changes in sociosexual attitudes, desires, and behavior in men. For this purpose, we have used data recruited during previous years of the COVID-19 pandemic about sociosexuality in Chilean men. In addition, we collected data about men’s sociosexuality during the COVID-19 pandemic in a similar sample of Chilean men. We set out the following predictions, which in turn are detailed in Table 1 in terms of the interplay between theoretical models.

**Fig. 1:**
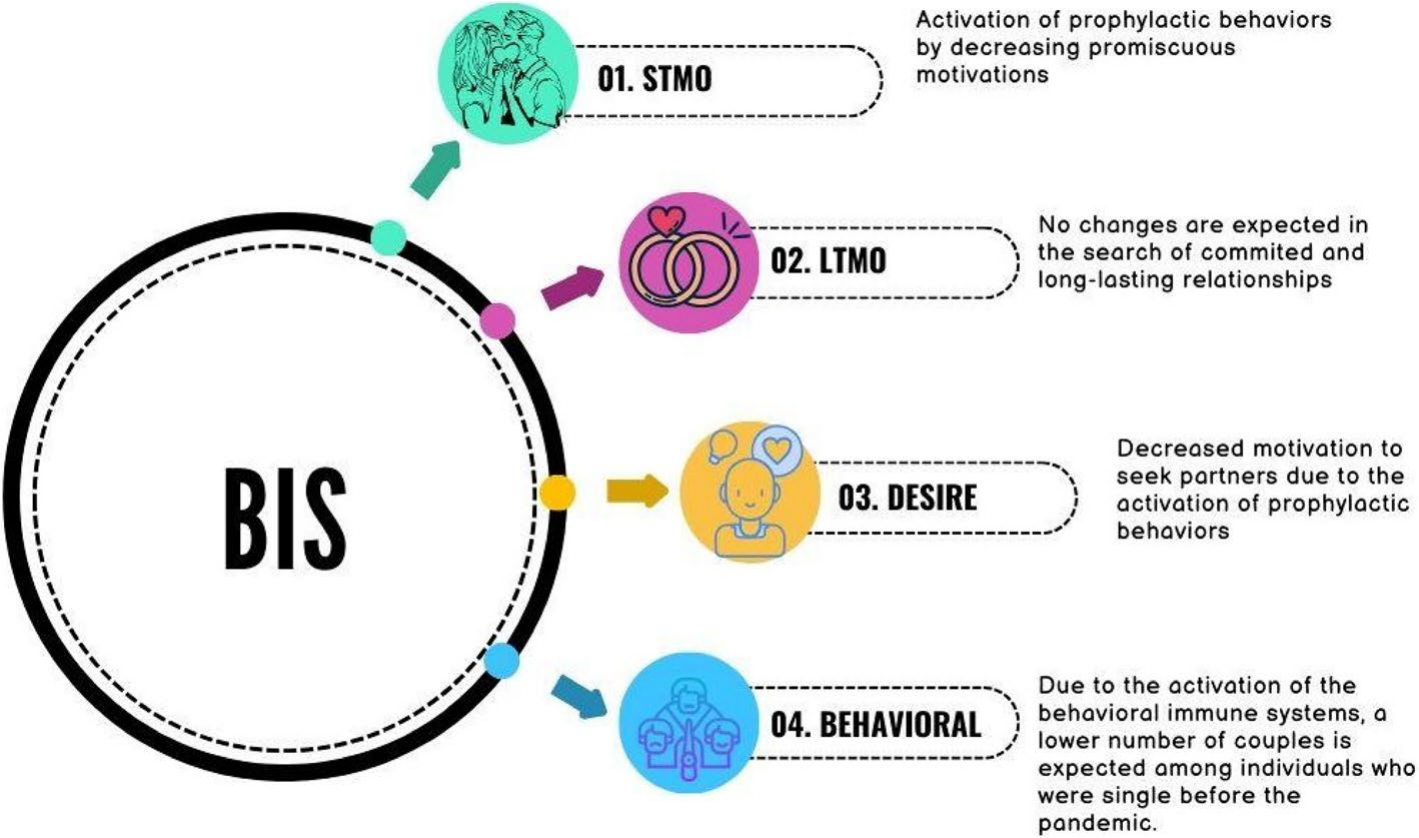
Relationship between behavioral immune system predictions and dimensions of sociosexuality. Note: BIS (behavioral immune system); LTMO (Long term mating orientation); STMO (short term mating orientation); sociosexual desire; Behavioral (behavioral dimension of sociosexuality

1. First, according to the behavioral immune system, we expect higher levels of short-term mating orientation reported before the pandemic compared to levels reported during it in single and paired individuals.
2. Second, since the prediction from the behavioral immune system is related to the decrease in seeking multiple mating partners, we do not expect any change in levels of long-term mating orientation in both single and paired individuals. However, given that there is no clear evidence for this prediction, we will test changes in this dimension in an exploratory manner.
3. Third, we expect higher levels of sociosexual desire reported before the pandemic compared to levels reported during it in single and paired individuals.
4. And finally, for single individuals, we expect a higher number of last-year sexual partners before the pandemic than during it (Fig. 1).

**Table 1:** Predictions derived from the behavioral immune, sociosexuality dimensions affected and the results expected from the interplay between theories

	**Dimension of theoretical models included on predictions**		
Prediction	Behavioral immune system	Sociosexuality dimensions	Expected new understanding from the theoretical interplay between constructs
1	Activation of prophylactic behaviors by decreasing promiscuous motivations	STMO	Confirmation of the effect of the behavioral immune system over the decrease in short term mating orientation
2	No changes are expected in the search of committed and long-lasting relationships	LTMO	New understanding about the precision of the activation of behavioral immune system which does not include long term mating orientation
3	Decreased motivation to seek partners due to the activation of prophylactic behaviors	Sociosexual desire	Clarify that the effect of behavioral immune system includes a mental pattern of decrease of desires of short term mating
4	Due to the activation of the behavioral immune systems, a lower number of couples is expected among individuals who were single before the pandemic	Behavioral	Confirm that there is coherence between sexual behavior, short-term dimensions and sociosexual desire

*STMO* Short term mating orientation, *LTMO* Long term mating orientation

## Materials and methods

### Participants

The sample consisted of 879 men between 18 and 58 years (M = 24.63; *SD* = 6.43) from the central zone of Chile who participated in four different projects before and during the pandemic period. Prestudies 1 and 2, were collected during 2016 (prestudy 1, N = 217) and 2018 (prestudy 2, N = 246). These samples correspond to projects oriented to the study of several traits related to short and long-term orientation in men (please see: [10, 35]) and the role of attractiveness and competitive strategies to understand short-term orientation in men (please see: [12]). We use data that includes answers of both the Sociodemographic questionnaire and Multidimensional Sociosexual Orientation. Pandemic data was collected between November 2020 to November 2021 in an online study using Qualtrics® platform (panstudy online, N = 182) and in a face-to-face panstudy (panstudy face-to-face, N = 234). In the case of the face-to-face samples (both prestudies and one panstudy), the participants received a payment of $30,000 Chilean pesos for their participation. The participants of the face-to-face panstudy came to the laboratory in groups of six individuals to participate in a study of intergroup conflict (please see [32]). They answered the Multidimensional Sociosexual Orientation previous to the participation in the intergroup conflict study. We decided not to apply a survey of COVID 19 in face-to-face panstudy since this could lead to participant exhaustion. For the online data collection, they were paid $4,000 Chilean pesos for answering the Sociosexual Orientation Inventory. In this sample we included the survey related to COVID-19. For the online panstudy, participants enrolled in a virtual lab and received a link that took them to the Qualtrics platform where they completed the aforementioned instruments. The team of scientists in all data collection processes, including prestudies 1 and 2 and panstudies face-to-face and online, pertains to the same group (Laboratorio de Comportamiento Animal y Humano, LABCAH) and were the same people. All the studies were performed in Spanish language. We refer to the data collected during the pandemic as panstudy to differentiate it from the data obtained prior to this period.

From the 879 participants, 3 failed to answer STMO scale, 4 failed to answer LTMO scale, 4 failed to report their age, 1 failed to report his relationship status and 61 failed to report weekly frequency of sexual fantasies.

There were no exclusion criteria to participate but to be a person self identified as a man over 18 years old. All participants were recruited through social networks and databases from previous projects. Questionnaires were answered at the first stage of all studies. Following this stage, participants played several economic games and other psychometric test in line with the objectives of the main projects (explained in the first paragraph) that varied across studies (i.e., public good games and prisoner dilemma games or the fighting ability questionnaire and parenthood abilities test), except for the online pandstudy in which no economic games were played. For all studies, we aimed to recruit men from the general population.

### Ethics

The research was approved by the ethics committee of the Universidad de Playa Ancha (prestudies 1 and 2) and by the ethics committee of the Universidad del Desarrollo (panstudies 1 and 2; face-to-face and online) and it follows international standards such as the Declaration of Helsinki. In the case of the face-to-face samples (prestudies 1 and 2 and panstudy face-to face), they were given a copy of the informed consent. In the case of the panstudy online sample, they were given the possibility of downloading the document upon participation from the Qualtrics® platform. In all studies, to protect privacy and facilitate the reliability of questionnaire answers, each participant received a randomized number at the beginning of data collection, making it impossible to unify the participants' numbers with their respective identities.

### Instruments

Sociodemographic Questionnaire: Participants answered a sociodemographic questionnaire that included gender, age and relationship status (single or paired).

Multidimensional Sociosexual Orientation Inventory (SOI-M): The multidimensional version developed by Jackson and Kirkpatrick [21] was utilized in this study. This instrument comprises 25 items designed to assess individual predisposition toward committing to an intimate relationship. It evaluates attitudinal components related to both sexual and emotional intimacy, indicating a predisposition for establishing either short- or long-term relationships. Additionally, it includes a dimension that assesses the history of past relationships and a question regarding the frequency of sexual fantasies involving individuals other than one's partner. Attitudinal questions utilize 7-point Likert-type responses where 1 means extremely disagree, and 7 means extremely agree, while the behavioral questions consist of open-ended questions in which individuals must indicate the number of sexual partners.. The authors define the scale across three dimensions: Short-Term Mating Orientation (STMO), Long-Term Mating Orientation (LTMO), and Previous Sexual Behavior. The obtained Cronbach's α values for these dimensions are 0.95, 0.88, and 0.83, respectively, demonstrating the instrument's good reliability. For this study, the STMO and LTMO subscales were employed. Additionally, we included a question about the number of sexual partners in the last year and a question about the weekly frequency of sexual fantasies. This allowed us to independently evaluate sociosexual desire and sociosexual behavior, as the latter question does not load into the STMO, LTMO, or behavioral dimensions. We excluded questions about the number of casual and lifetime sexual partners, as our focus was on capturing changes in sociosexual behavior during the pandemic, and these questions encompass the entire lifespan. Furthermore, for the question regarding the number of sexual partners in the last year, we considered only those individuals who participated at least one year after the start of the pandemic. This decision was made to capture changes specifically within the pandemic context. In our sample, Cronbach's α values of 0.81 for STMO and 0.73 for LTMO were obtained, indicating good reliability of the instrument. We opted for this version of the SOI because it considers short- and long-term dimensions orthogonally, allowing us to capture the simultaneous deployment of both strategies in the same individual.

World Health Organization Survey: To measure the perception of the risk of contagion, we employed one question in the World Health Organization Survey “What do you think is your own chance of getting COVID-19?” that is answered in a 7-point Likert scale (i.e., 1 = extremely unlikely to 7 = extremely likely). This information is only available for individuals who participated in the online panstudy (N = 182). Individuals scoring from 4 to 7 were classified as having a high perception of risk of infection (N = 97), otherwise, they were classified as having a low perception of risk of infection (N = 71). Fourteen individuals failed to answer this question. This categorization was based on creating groups as equal as possible in size since the 4-point response is a middle point in the scale. If we had assigned the 4-point response to the low perception group we would have a more unequal distribution of 37 individuals in the high perception group and 131 in the low perception group.

### Data analysis

First, in order to account for potential differences within prestudies 1 and 2, and pandemic studies face-to-face and online, due to different approaches to gather the data in the pandemic samples and potential differences between studies in the prestudies samples (both face-to-face samples but belonging to different projects), we performed a t-test of independent samples. Whenever we did not find differences, we collapsed samples in two categories of prestudies (1 and 2) and panstudies (face-to-face and online) in order to compare them. However, if differences were detected between studies in the prepandemic context, we kept them as separate categories of study 1 and study 2. Similarly, if differences were detected between studies in the pandemic context, we kept them as separate categories of online and face-to-face panstudies.

To test our predictions, we fitted four general linear models. In the first model, the dependent variable was the mean score of STMO, and the independent variables were the pandemic context (prestudies vs panstudies, or separating between studies when necessary), the relationship status (single or paired) and we controlled for age. We tested first for main effects and then we included the interaction between context and relationship status to test whether the effect of the pandemic context was different according to the relationship status. In the second, third and fourth models, we employed the same procedure but changing the dependent variables. In the second model the dependent variable was the mean score of LTMO, in the third model the dependent variable was the weekly frequency of sociosexual fantasies with uncommitted partners and in the fourth model the dependent variable was the number of last-year sexual partners. The interaction term between pandemic context and relationship status was included in prediction 1, 2 and 3 as an exploratory analysis since we specifically predicted only a main effect of the pandemic context.

Considering the importance of the perception of the risk of contagion in the activation of the behavioral immune system, but given that we only captured that information during the panstudy online, we performed an additional set of analysis considering a restricted data sample in which we differentiate three different contexts: individuals in the prepandemic period, individuals with a high perception of contagion risk during the pandemic period and individuals with low perception of contagion risk during the pandemic period. We performed the same analysis as described for the full sample as a means of control for the effect of the perception of risk of contagion in changes of sociosexuality.

Post-hoc comparisons using Sidak correction followed whenever a significant effect was found in a categorical variable with more than two factors. Data was not transformed and only missing data was not considered, that is, we did not exclude potential outliers from our sample as we did not have theoretical reasons to exclude them. To establish a baseline, we considered all available data related to previous research projects we collected before the pandemic and aimed to collect as much data as possible during the pandemic. Since the data was already collected, we performed a post hoc sensitivity analysis to assess the minimum effect size that we can detect considering our sample size and data analysis design. Regarding the full data sample and the most complex model (sociosexual fantasies with 8 predictors), and considering an α = 0.05, a power of 0.80 and a available sample size of 815 the minimum detectable effect size is f^2^ = 0.019 which is considered a small effect size [6]. Regarding the reduced data sample and the most complex model (sociosexual fantasies with 8 predictors), and considering an α = 0.05, a power of 0.80 and an available sample size of 568 the minimum detectable effect size is f^2^ = 0.027 which is considered a small effect size [6]. Power analyses were performed with G*Power3.1.9.6 [11]. All the remaining analyses were performed with IBM SPSS 25 and we set up the level of significance at α = 0.05.

## Results

### Descriptive analysis

Table 2 presents the descriptive statistics for the variables age, short-term mating orientation and long-term mating orientation, sex fantasies, and number of partners in the last year.

**Table 2:** Descriptive statistics

Variable	N	M ± *SD*
Age	875	24.63 ± 6.43
STMO	876	4.36 ± 1.30
LTMO	874	5.20 ± 1.22
SEX Fantasies	818	2.50 ± 2.21
N Partners	776	1.57 ± 1.90

Descriptive Statistics (M ± *SD*) of the total sample for the variables, age, short-term mating orientation (STMO) and long-term mating orientation (LTMO), Sexual fantasies (SEX Fantasies), Number of partners in the last year (N Partners)

### Differences between prestudies 1 and 2

We did not find significant differences between responses in the prestudies 1 and 2 in STMO (M_study1_ = 4.43, SD_study1_ = 1.35; M_study2_ = 4.45, SD_study2_ = 1.42;*t* = -0.17, df = 458, p = 0.863, d = -0.016), LTMO (M_study1_ = 5.41, SD_study1_ = 1.29; M_study2_ = 5.35, SD_study2_ = 1.42; *t* = 0.51, df = 456, p = 0.610, d = 0.048) and number of sexual partners last year (M_study1_ = 1.68, SD_study1_ = 1.95; M_study2_ = 1.59, SD_study2_ = 1.79; *t* = 0.48, df = 457, p = 0.629, d = 0.045). However, individuals from study 1 reported higher frequency of sexual fantasies than individuals from study 2 (M_study1_ = 2.87, SD_study1_ = 2.16; M_study2_ = 2.32, SD_study2_ = 2.20; *t* = 2.51, df = 401, p = 0.012, d = 0.251).

### Differences between panstudies online and face-to-face

We did not find significant differences between responses in the panstudy online sample compared to panstudy face-to-face in STMO (M_online_ = 4.34, SD_online_ = 1.44; M_face-to-face_ = 4.26, SD_face-to-face_ = 1.00;*t* = 0.63, df = 414, p = 0.529, d = 0.062), and the number of sexual partners last year (M_online_ = 1.41, SD_online_ = 2.10; M_face-to-face_ = 1.56, SD_face-to-face_ = 1.77; *t* = -0.67, df = 315, p = 0.501, d = -0.076). However, individuals in the online sample reported higher LTMO compared to face-to-face sample (M_online_ = 5.29, SD_online_ = 1.39; M_face-to-face_ = 4.79, SD_face-to-face_ = 0.86; *t* = 4.50, df = 414, p < 0.001, d = 0.444) and individuals in the online sample reported less frequency of sexual fantasies compared to face-to-face sample (M_online_ = 1.76, SD_online_ = 2.13; M_face-to-face_ = 2.89, SD_face-to-face_ = 2.18; *t* = -5.29, df = 413, p < 0.001, d = -0.524).

### Effect of the pandemic and relationship status over sociosexuality (full data set)

Regarding our first prediction (Table 3), we did not find a significant effect of the pandemic context as a main effect (*F* (1, 867) = 1.35, p = . 245, η^2^ = . 002) neither in interaction with relationship status (*F* (1, 866) = 1.08, p = . 299, η^2^ = . 001) on levels of STMO. We found a significant effect of the relational status (*F* (1, 867) = 11.68, p = 0.001, η^2^ = 0.013), being single individuals higher in STMO (M = 4.53, *SE* = 0.06) compared to paired individuals (M = 4.23, *SE* = 0.06).

**Table 3:** Coefficients and standard errors for the models with the full data regarding short-term mating orientation, and number of last-year sexual partners

	STMO (Prediction 1)	N Partners (Prediction 4)
Intercept	4.23*** (.21)	.51 (.34)
Context = 0	.11 (.09)	.31* (.15)
RS = 0	.30** (.09)	.23 (.14)
Age	< -.01 (.01)	.03** (.01)
Context = 0 * RS = 0	.18 (.18)	.09 (.28)

Short-term mating orientation (STMO), Number of partners in the last year (N Partners), Context = 0: Prepandemic studies, RS = 0: Single individuals. Standard errors are shown within parenthesis. First, we fit the model only with the main effects (coefficients shown in the table) and then we included the interaction term

^*^ *p* < .05, ** *p *< .01, *** *p* < .001

**Fig. 2:**
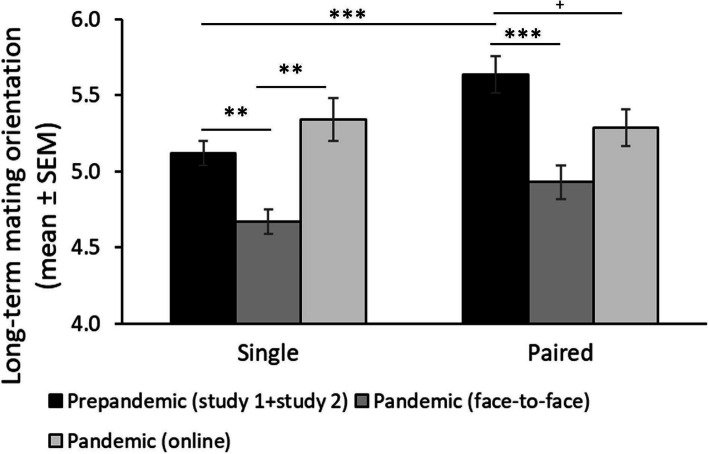
Long term sociosexuality. Note: Mean and standard errors in long-term sociosexuality across periods and according to the relationship status. ** p < .01, *** p < .001, + .05 < p < .60

Regarding our second prediction (see Table 4 and Fig. 2), we found a significant interaction between relationship status and the pandemic context (*F* (2, 862) = 3.83, p = 0.022, η^2^ = 0.009; Fig. 2) on levels of LTMO. To interpret this interaction, first, we focus on differences between contexts (prepandemic vs online panstudy vs face-to-face panstudy) according to relationship status (paired vs single). In this sense, paired individuals showed higher levels of LTMO in prepandemic context (study 1 and study 2) (M = 5.64, *SE* = 0.08) compared to face-to-face panstudy (M = 4.93, *SE* = 0.11; mean differences = 0.71, *SE* = 0.13, *p* < 0.001) and a statistical trend was found for the online panstudy (M = 5.29, *SE* = 0.12; mean differences = 0.36, *SE* = 0.15, *p* = 0.050). Paired individuals in the face-to-face and online panstudies did not differ in LTMO (mean differences = 0.36, *SE* = 0.16, p = 0.074). Single individuals showed higher levels of LTMO during prepandemic context (M = 5.12, *SE* = 0.08) compared to face-to-face panstudy (M = 4.67, *SE* = 0.12; mean differences = 0.46, *SE* = 0.14, p = 0.003) but no differences were found with online panstudy (M = 5.34, *SE* = 0.14; mean differences = -0.21, *SE* = 0.16, p = 0.459). Single individuals in the face-to-face panstudy reported lower levels of LTMO than single individuals in the online panstudy (mean differences = -0.67, *SE* = 0.18, *p* < 0.001). If we focus on differences between single and paired individuals according to pandemic context, we found that paired individuals reported higher LTMO in the prepandemic context compared to single ones (mean differences = 0.52, *SE* = 0.11, *p* < 0.001), but no differences between paired and singles individuals were found during the face-to-face panstudy (mean differences = 0.26, *SE* = 0.16, *p* = 0.092) and online panstudy (mean differences = -0.05, *SE* = 0.17, p = 0.769).

**Table 4:** Coefficients and standard errors for the models with the full data regarding long-term mating orientation

	LTMO
Intercept	5.56*** (.40)
Context = 0	.10* (.11)
Context = 1	-.49*** (.12)
RS = 0	.33*** (.08)
Age	.03 (.02)
Context = 0 * RS = 0	-.57** (.21)
Context = 1 * RS = 0	-.32 (.24)

Long-term mating orientation (LTMO), Context = 0: Prepandemic studies, Context = 1: Pandemic face-to-face study, RS = 0: Single individuals. Standard errors are shown within parenthesis. First, we fit the model only with the main effects (coefficients shown in the table) and then we included the interaction term

^*^ *p* < .05, ** *p* < .01, *** *p* < .001

**Fig. 3:**
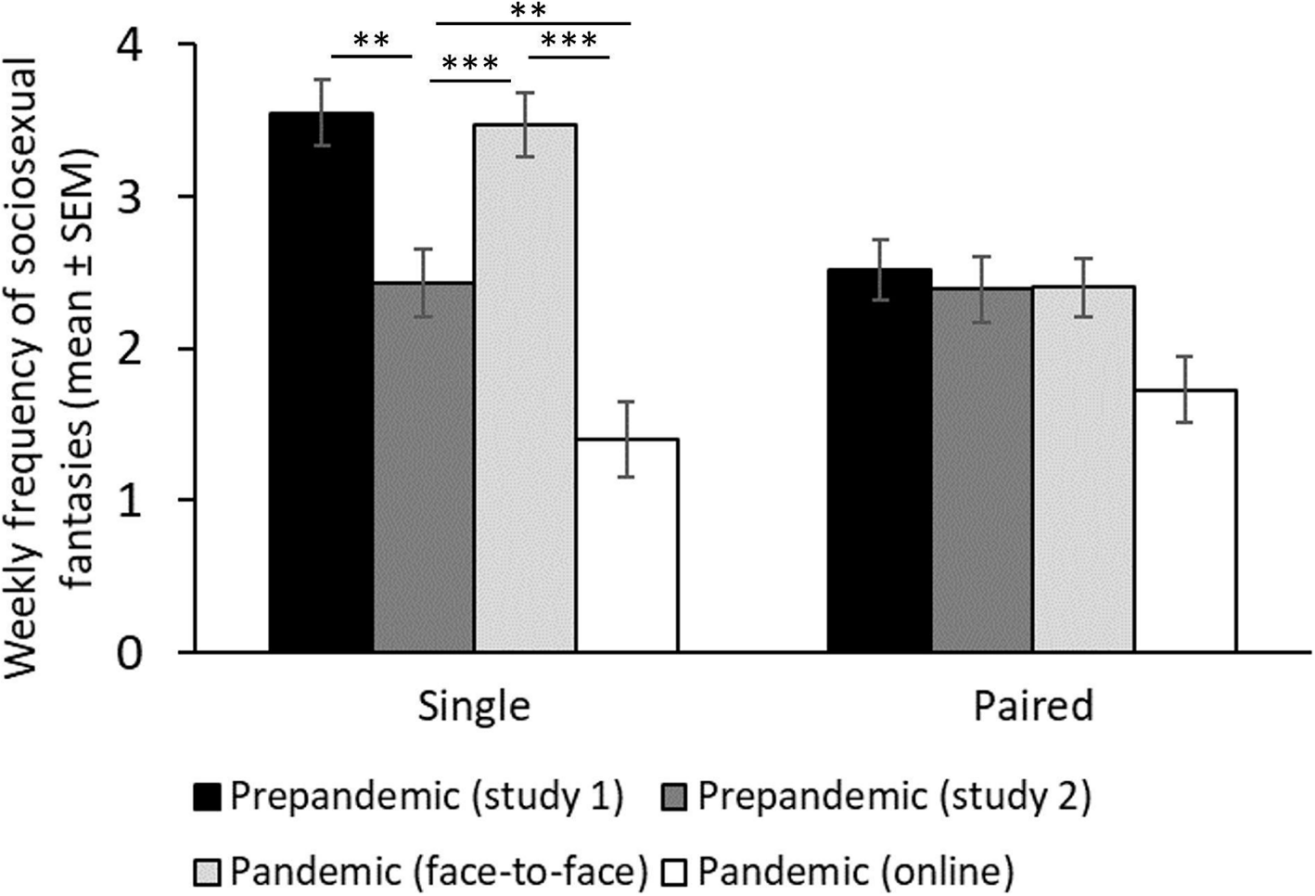
Sociosexual desire. *Note:* Mean and standard errors in sociosexual desire (measured as weekly frequency of sexual fantasies) across periods and according to the relationship status. ** *p* < .01, *** *p* < .001

Regarding our third prediction (see Table 5, and Fig. 3), we found a significant interaction between relationships status and the pandemic context (*F* (3, 806) = 5.47, p = 0.001, η^2^ = 0.020; Fig. 3) on weekly frequency of sociosexual fantasies. To interpret this interaction, first, we focus on differences between contexts (prestudy 1 vs prestudy 2 vs online panstudy vs face-to-face panstudy) according to relationship status (paired vs single). Single individuals showed higher frequency of sociosexual fantasies during prestudy 1 (M = 3.55, *SE* = 0.22) compared to individuals during the prestudy 2 (M = 2.43, *SE* = 0.22, mean differences = 1.13, *SE* = 0.31, p = 0.002) and compared to individuals during the online panstudy (M = 1.40, *SE* = 0.25, mean differences = 2.15, *SE* = 0.34, p < 0.001). However, no differences were found with the face-to-face panstudy (M = 3.47, *SE* = 0.21; mean differences = 0.09, *SE* = 0.30, p = 1.000). In addition, single individuals reported higher frequency of sociosexual fantasies during the prestudy 2 compared to individuals during the online panstudy (mean differences = 1.03, *SE* = 0.34, p = 0.014) but lower compared to individuals in the face-to-face panstudy (mean differences = -1.04, *SE* = 0.30, p = 0.004). Finally, single individuals reported higher frequency of sociosexual fantasies during the face-to-face panstudy compared to the online panstudy (mean differences = 2.07, *SE* = 0.32, p < 0.001). When considering paired individuals, we found that individuals did not differ in the frequency of sociosexual fantasies across studies (prestudy 1 vs prestudy 2: mean differences = 0.12, *SE* = 0.30, p = 0.999; prestudy 1 vs face-to face panstudy: mean differences = 0.12, *SE* = 0.27, p = 0.999; prestudy 1 vs panstudy online: mean differences = 0.79, *SE* = 0.30, p = 0.051; prestudy 2 vs panstudy face-to face: mean differences < -0.01, *SE* = 0.29, p = 1.000; prestudy 2 vs panstudy online: mean differences = 0.67, *SE* = 0.31, p = 0.187; panstudy face-to face vs pandemic online study: mean differences = 0.67, *SE* = 0.29, p = 0.115). If we focus on differences between single and paired individuals according to pandemic context, we found that single individuals reported higher frequency of sociosexual fantasies compared to paired ones in the prestudy 1 (mean differences = 1.04, *SE* = 0.29, p < 0.001) and in panstudy face-to-face (mean differences = 1.07, *SE* = 0.28, p < 0.001). No differences were found between single and paired individuals in the prestudy 2 (mean differences = 0.03, *SE* = 0.31, p = 0.922) and in the online pandemic study (mean differences = -0.33, *SE* = 0.31, p = 0.307).

**Table 5:** Coefficients and standard errors for the model with the full data regarding frequency of weekly sexual fantasies

	Sexual fantasies
Intercept	1.70*** (.36)
Context = 0	.10 (.21)
Context = 1	-.50* (.21)
Context = 2	-1.30*** (.22)
RS = 0	.51** (.15)
Age	.04** (.01)
Context = 0 * RS = 0	-.03 (.41)
Context = 1 * RS = 0	-1.04* (.42)
Context = 2 * RS = 0	-1.39** (.42)

Context = 0: Prepandemic study 1, Context = 1: Prepandemic study 2 Context = 2: Pandemic face-to-face study, RS = 0: Single individuals. Standard errors are shown within parenthesis. First, we fit the model only with the main effects (coefficients shown in the table) and then we included the interaction term

^*^ *p* < .05, ** *p* < .01, *** *p* < .001

**Fig. 4:**
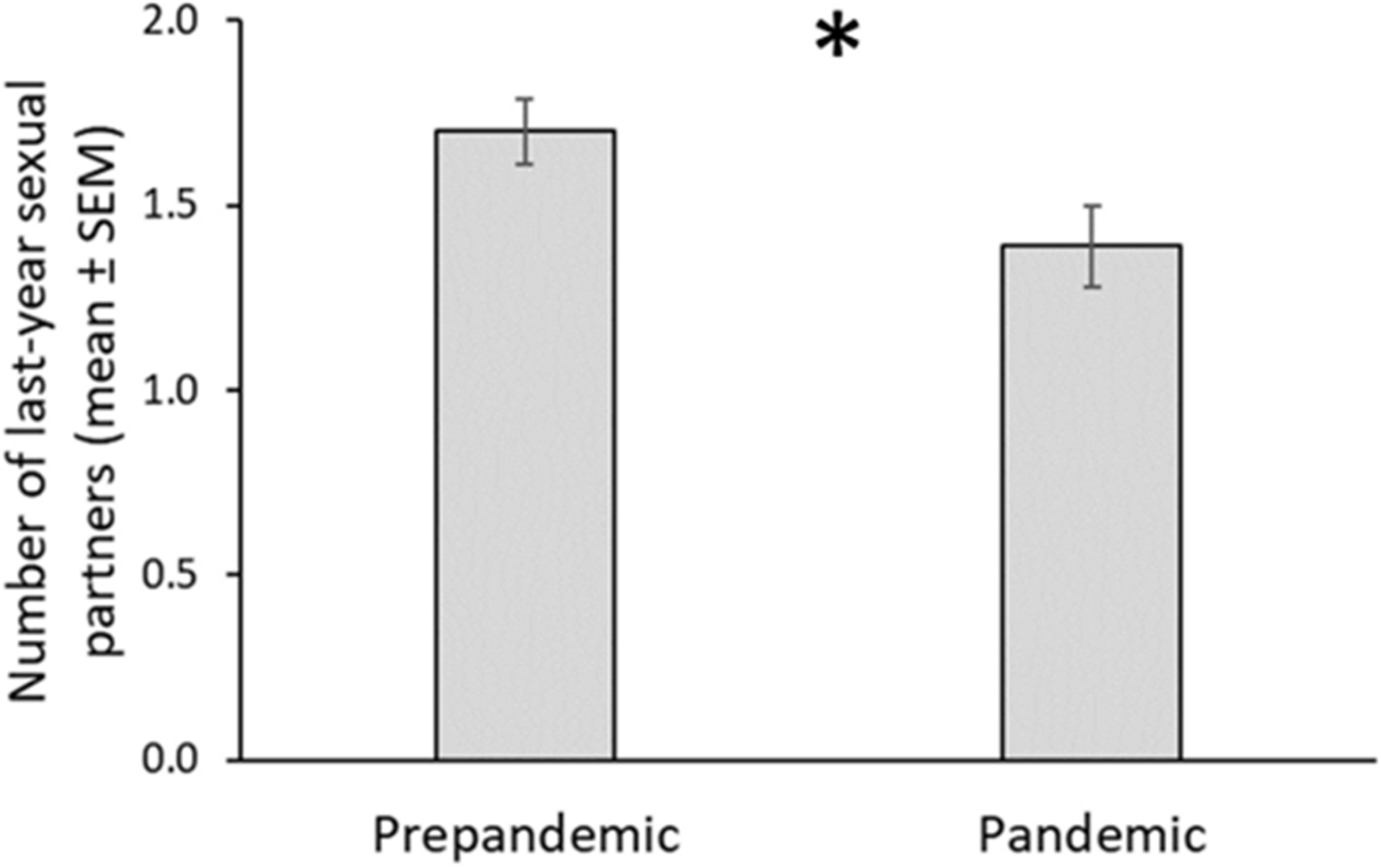
Number of last year sexual partners. *Note:* Mean and standard errors in sociosexual behavior (measured as number of last-year sexual partners) across periods. * *p* < .05

Regarding our fourth prediction (Table 3), we did not find a decreased number of last year sexual partners in single individuals during the pandemic period compared to pre pandemic period as the interaction between pandemic context and relationship status was not significant (*F* (1, 766) = 0.10, p = 0.752, η^2^ < 0.001). However, we found a main effect of the pandemic context (*F* (1, 767) = 4.28, p = 0.039; η^2^ = 0.006; Fig. 4). Individuals before the pandemic period reported higher numbers of last-year sexual partners (M = 1.70, *SE* = 0.09) compared to the pandemic period (M = 1.39, *SE* = 0.11) regardless of their relationship status. The main effect of relationship status only reached a statistical trend (F(1, 767) = 2.78, p = 0.096, η^2^ = 0.004). Single individuals reported a higher number of last-year sexual partner (M = 1.66, *SE* = 0.10) compared to paired individuals (M = 1.43, *SE* = 0.10).

### Effect of the perception of risk of contagion over sociosexuality (reduced dataset)

When considering the degree of perception of risk of contagion during the pandemic (panstudy online) we found the following results for each of our predictions.

For our first prediction (Table 6), we failed to find an effect of the context (*F* (2, 619) = 0.37, p = 0.691, η^2^ = 0.001). Individuals before the pandemic period did not reported higher levels of STMO (M = 4.42, *SE* = 0.07) compared to individuals with a high perception of risk of contagion during the pandemic period (M = 4.30, *SE* = 0.16; mean differences = 0.12, *SE* = 0.18, p = 0.864) nor to individuals with a low perception of risk of contagion during the pandemic period (M = 4.48, *SE* = 0.17; mean differences = -0.06, *SE* = 0.19, p = 0.985). Similar to the model with full data, we found an effect of the relationship status (*F* (1, 619) = 5.36, p = 0.021, η^2^ = 0.009). Finally, we did not find an interaction between the pandemic context and relationships status (*F* (2, 617) = 1.61, p = 0.201, η^2^ = 0.005).

**Table 6:** Coefficients and standard errors for the models with the reduced data regarding short-term mating orientation, long-term mating orientation and number of last-year sexual partners

	STMO (Prediction1)	LTMO (Prediction 2)	N Partners (Prediction 4)
Intercept	4.50*** (.24)	5.53*** (.22)	.50 (.33)
Context = 0	.06 (.19)	< -.01 (.17)	-.02 (.26)
Context = 1	-.12 (.18)	-.25 (.16)	-.76** (.24)
RS = 0	.26* (.09)	-.37*** (.11)	.34* (.16)
Age	-.01 (.01)	< .01 (.01)	.04** (.01)
Context = 0 * RS = 0	-.47 (.31)	.60 (.29)	.12 (.44)

Short-term mating orientation (STMO), Long-term mating orientation (LTMO), Number of partners in the last year (N Partners), Context = 0: Prepandemic studies, RS = 0: Single individuals. Standard errors are shown within parenthesis. First, we fit the model only with the main effects (coefficients shown in the table) and then we included the interaction term

^*^ *p* < .05, ** *p* < .01, *** *p* < .001

Regarding LTMO (Table 6), we did not find a main effect of the context (*F* (2, 617) = 1.18, p = 0.307, η^2^ = 0.004). Individuals before the pandemic period did not reported higher levels of LTMO (M = 5.40, *SE* = 0.06) compared to individuals with a high perception of risk of contagion during the pandemic period (M = 5.15, *SE* = 0.15; mean differences = 0.25, *SE* = 0.16, p = 0.405) nor to individuals with a low perception of risk of contagion during the pandemic period (M = 5.39, *SE* = 0.16; mean differences < 0.01, *SE* = 0.17, p = 1.000).

Similar to the model with full data, we found an effect of the relationship status (*F* (1, 617 = 12.25, p < 0.001, η^2^ = 0.019). Finally, we did not find an interaction between the context and relationship status (*F* (2, 615) = 2.61, p = 0.074, η^2^ = 0.008).

**Fig. 5:**
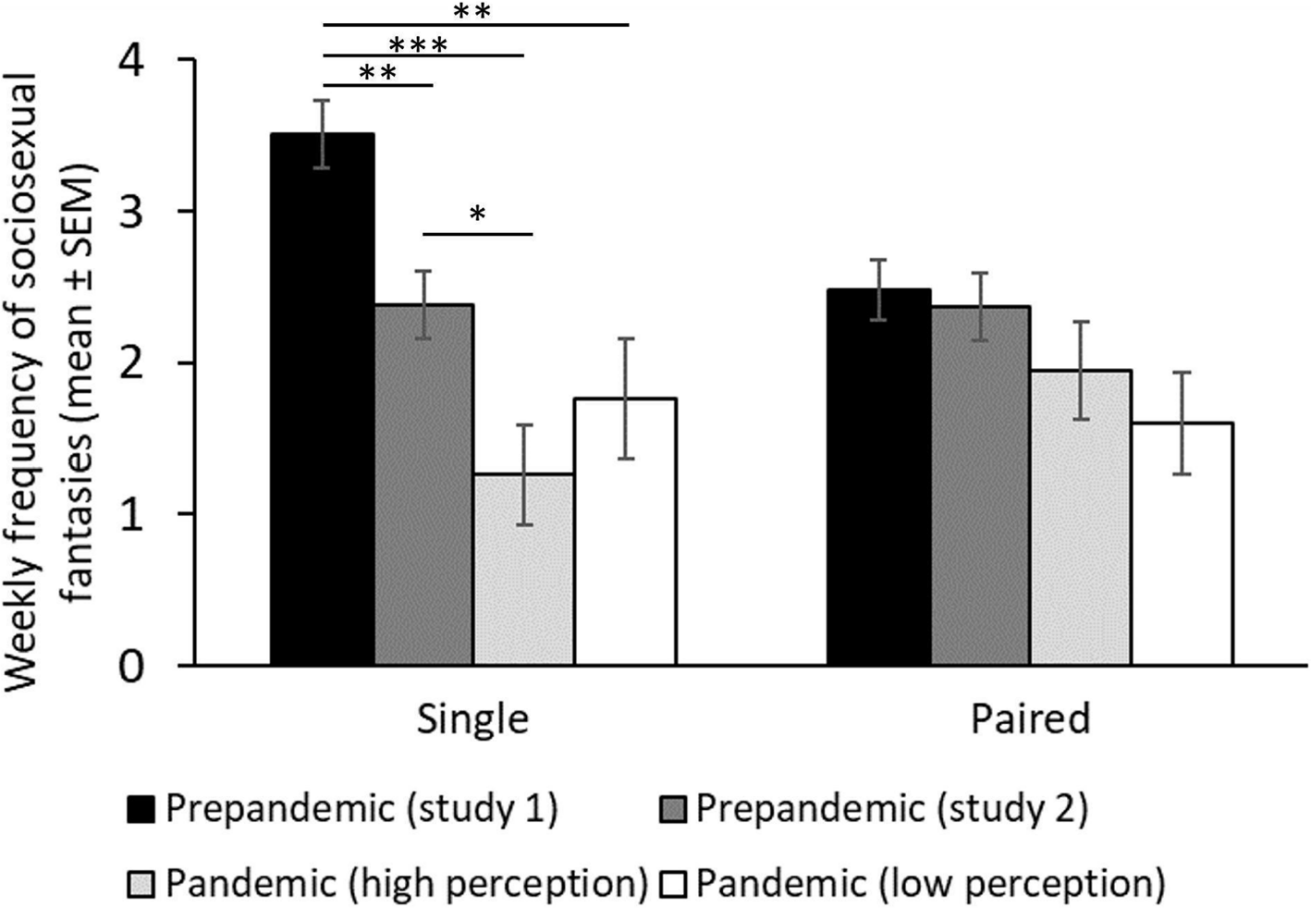
Sociosexual desire. Note*:* Mean and standard errors in sociosexual desire (measured as weekly frequency of sexual fantasies) across periods, categories of risk perceptions, and according to the relationship status. * p < .05, ** p < .01, *** p < .001, + .05 < p < .60

Regarding weekly frequency of sociosexual fantasies (Table 7), we found an interaction between the context and relationships status (*F* (3, 559) = 4.06, p = 0.007, η^2^ = 0.021; Fig. 5). To interpret this interaction, first, we focus on differences between contexts (prestudy 1 vs prestudy 2 vs high risk perception vs low risk perception) according to relationship status (paired vs single). Single individuals showed higher frequency of sociosexual fantasies during prestudy 1 (M = 3.51, *SE* = 0.22) compared to individuals during the prestudy 2 (M = 2.38, *SE* = 0.22, mean differences = 1.13, *SE* = 0.31, p = 0.002), individuals with high perception of contagion risk during the pandemic (M = 1.26, *SE* = 0.33; mean differences = 2.25, *SE* = 0.41, p < 0.001) and individuals with low perception of contagion risk during the pandemic (M = 1.76, *SE* = 0.40; mean differences = 1.75, *SE* = 0.46, p = 0.001). Single individuals showed higher frequency of sociosexual fantasies during prestudy 2 compared to individuals with high perception of contagion risk during the pandemic (mean differences = 1.12, *SE* = 0.41, p = 0.037) but no to individuals with low perception of contagion risk during the pandemic (mean differences = 0.62, *SE* = 0.46, p = 0.694). Single individuals with high and low perception of contagion risk during the pandemic did not differ in their frequency of sexual fantasies (mean differences = -0.50, *SE* = 0.51, p = 0.912). Regarding paired individuals, we found that individuals did not differ in the frequency of sexual fantasies across studies (prestudy 1 vs prestudy 2: mean differences = 0.10, *SE* = 0.30, p = 1.000; prestudy 1 vs high perception of contagion risk during the pandemic (panstudy online): mean differences = 0.53, *SE* = 0.38, p = 0.670; prestudy 1 vs low perception of contagion risk during the pandemic (panstudy online): mean differences = 0.88, *SE* = 0.39, p = 0.143; prepandemic study 2 vs high perception of contagion risk during the pandemic: mean differences = 0.43, *SE* = 0.39, p = 0.857; prestudy 2 vs low perception of contagion risk during the pandemic: mean differences = 0.78, *SE* = 0.40, p = 0.286; high vs low perception of contagion risk during the pandemic (panstudy online): mean differences = 0.35, *SE* = 0.45, p = 0.969). If we focus on differences between single and paired individuals according to pandemic context, we only found differences in prestudy 1 since single individuals reported higher frequency of sociosexual fantasies compared to paired ones (mean differences = 1.03, *SE* = 0.30, p < 0.001). No differences were found between single and paired individuals in the prestudy 2 (mean differences = 0.01, *SE* = 0.32, p = 0.984) and in the individuals with high (mean differences = -0.69, *SE* = 0.44, p = 0.119) and low (mean differences = 0.16, *SE* = 0.52, p = 0.757) perception of contagion risk during the pandemic.

**Table 7:** Coefficients and standard errors for the model with the full data regarding frequency of weekly sexual fantasies

	Sexual fantasies
Intercept	1.57*** (.40)
Context = 0	-.70* (.31)
Context = 1	-.76* (.30)
Context = 2	.58** (.22)
RS = 0	.30 (.18)
Age	.03 (.02)
Context = 0 * RS = 0	.15 (.61)
Context = 1 * RS = 0	-.69 (.54)
Context = 2 * RS = 0	1.03* (.43)

Context = 0: Prepandemic study 1, Context = 1: Prepandemic study 2 Context = 2: high perception of contagion risk in pandemic studies, RS = 0: Single individuals. Standard errors are shown within parenthesis

^*^ *p* < .05, ** *p* < .01, *** *p* < .001

**Fig. 6:**
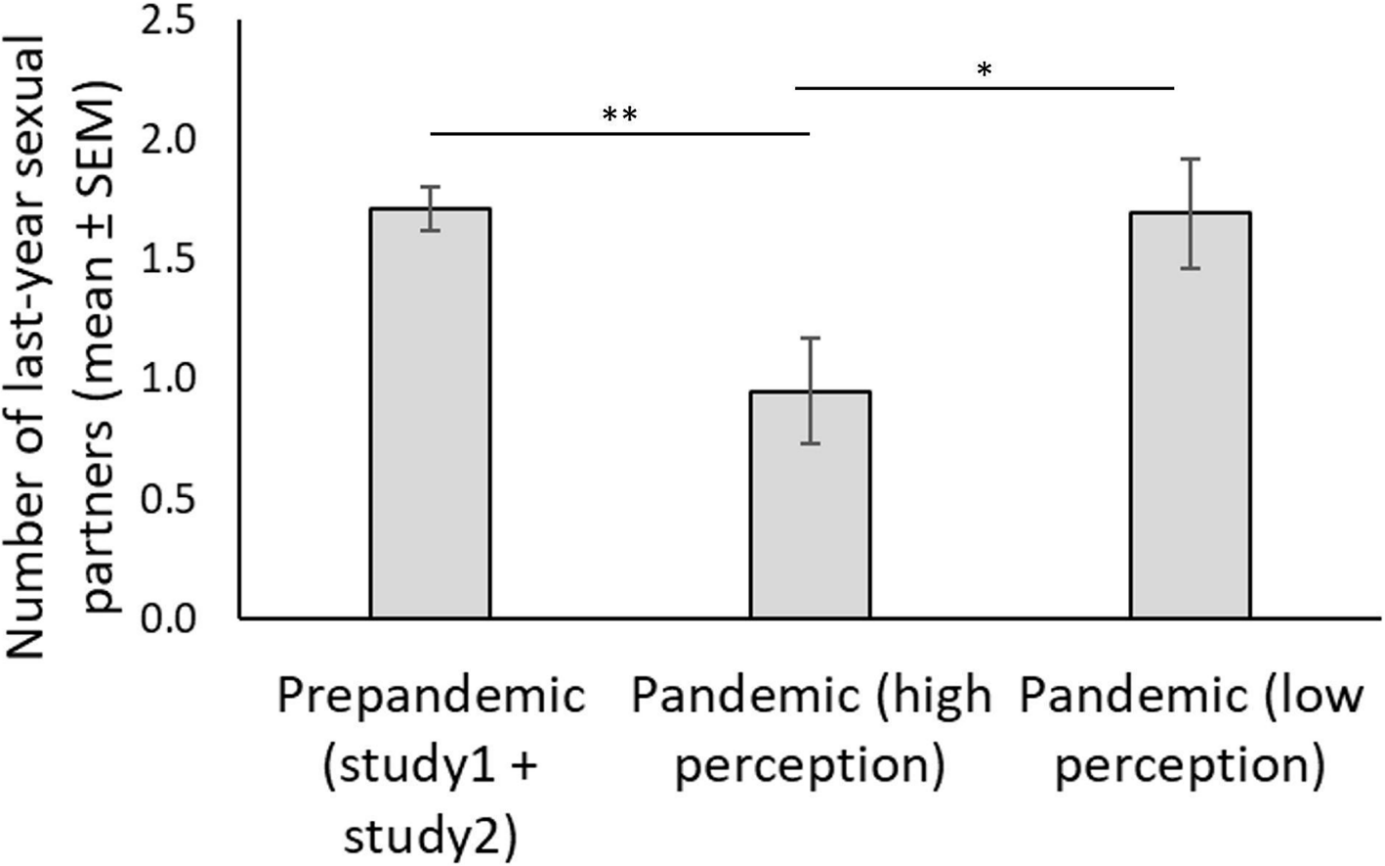
Number of last year sexual partners. Note: Mean and standard errors in sociosexual behavior (measured as number of last-year sexual partners) across periods and risk perceptions. ** p < .01, *** p < .001

Finally, regarding the number of last-year sexual partners (Table 6), we found a main effect of the context (*F* (2, 617) = 5.12, p = 0.006, η^2^ = 0.01; Fig. 6). Individuals during the prepandemic period reported higher number of last-year sexual partners (M = 1.71, *SE* = 0.09) than individuals with a high perception of contagion risk during the pandemic period (M = 0.95, *SE* = 0.22; mean differences = 0.76, *SE* = 0.24, p = 0.006) but no differences were found with individual low in perception of contagion risk during the pandemic period (M = 1.69, *SE* = 0.23; mean differences = 0.02, *SE* = 0.25, p = 1.00). In addition, during the pandemic period individuals with low perception of contagion risk reported higher number of last-year sexual partners compared to individual with high perception in contagion risk (mean differences = 0.74, *SE* = 0.31, p = 0.048). Similar to the model with full data, we found an effect of the relationship status (F (1, 617) = 4.82, p = 0.029, η^2^ = 0.008). Finally, we did not find an interaction between the context and relationships status (F (2, 617) = 0.38, p = 0.685, η^2^ < 0.001).

## Discussion

According to the behavioral immune system, humans may limit their social interactions due to the risk of contracting diseases (Schaller, et al., 2011). In our study, we tested the hypothesis that men's sociosexual attitudes, desires and behaviors would decrease during the pandemic compared to pre-pandemic periods. Our results partially supported our predictions regarding sociosexual desire and behavior during the pandemic. Surprisingly, we also found a decrease in long-term mating orientation during the pandemic period. Additionally, we analyzed the data from the online sample, considering the effects of self-perception of contagion risk (high/low), which would reflect the activation of the behavioral immune system. In a similar vein, we only found favorable evidence regarding desire and sociosexual behavior.

Mating, and more concretely sexual promiscuity, is a risky behavior, i.e., sexual contact and physical proximity expose individuals to sexual and non-sexual pathogens [39, 41]. For these reasons, an increase in pathogen prevalence like that occurring during the COVID-19 pandemic is expected to reduce levels of short-term mating orientation as this sociosexual attitude is linked to promiscuous mating behavior (e.g., [27]). Conversely, our results do not show that men displayed lower levels of short-term mating orientation during the pandemic outbreak than data collected before the pandemic. We found an effect of relational status, with singles reporting higher levels of STMO. This is consistent with evidence showing that single men show higher levels of STMO when compared to heterosexual men during the pandemic [42]. Regarding the perception of contagion risk and following previous evidence (e.g., [9]), we expected to find a decline in STMO in those individuals during the pandemic that showed high levels of perception of contagion risk. However, we did not find any effect in the expression of STMO. That is, we failed to find differences between individuals in the prepandemic period with individuals during pandemic with high perception of risk contagion. Overall, our results do not support our prediction suggesting that the expression of STMO seems not to be affected in the context of a pandemic. In this case, STMO may reflect a more stable trait that is more influenced by additive genetic factors compared to environmental factors [3, 24, 47]. In this sense, it has been established that women would be more sensitive to contextual changes (i.e., presence of pathogens) than men who would maintain their STMO in a stable manner, as a result of their genetic quality. Westerlund et al., [47], found that sociosexual behavior and attitudes were influenced by genetic factors, which supports the hypothesis that differences in men's partner value are related to the probability of participating in casual sexual encounters, this in turn is influenced by genetic factors, which would explain why the STMO is stable despite the contextual changes experienced during the pandemic period. This, unlike women who relax their STMO in environments where pathogens are present [40], favoring a preference for men with better genetic quality (i.e., more attractive), it seems that men oriented toward the short-term maintain a stable attitude and desires independent of changes in context.

Regarding our second prediction, given that long-term-oriented individuals are less likely to exhibit promiscuous and risky behaviors [44], we expected that the COVID-19 pandemic would not affect their expression. However, contrary to what we predicted, we found that individuals in the prepandemic period showed higher levels of LTMO than individuals during the pandemic, but only for the panstudy face-to-face sample. For single individuals, we also found a difference between online and face-to-face studies in the context of a pandemic. In this sense, both paired and single individuals showed a greater long-term orientation in the prepandemic period compared to face-to-face study during the pandemic. This decline occurs in both single and paired individuals. Still, while single individuals were less long-term oriented in the prepandemic context compared to paired individuals, paired and single individuals did not differ in long-term mating orientation during the panstudy face-to-face and online. This result suggests that the pandemic context affected more paired individuals in their expression of LTMO. Several reasons can explain this result in long-term strategies. First, single men who are oriented toward long-term mating still need to look for a new partner to establish a committed relationship. This requires a certain degree of intimacy at some point with the consequent risk of contagion. So, the pandemic may not have affected short-term mating orientation but long-term mating orientation [41]. However, this does not explain why non-single individuals also reduced their long-term mating orientation during the pandemic. A second reason lies in the changes at the economic level and the recession that we are experiencing worldwide [2, 41] and considering that socioeconomic status is an important trait that affects the expression of relationships long-term mating orientation in men [10], our results could be a consequence of a reduction in this orientation during the pandemic context.

On the other hand, when we analyze the perception of the risk of contagion during the pandemic period, we do not find an effect of context (pre- and pandemic period). Individuals did not report higher levels of LTMO in the pre-pandemic period compared to individuals who showed a high or low perception of contagion risk. This result suggests that the low levels of LTMO during the pandemic period were manifested only in the face-to-face sample.

Regarding our third prediction, we expected a reduction in sociosexual desire, measured through the weekly frequency of sexual fantasies, during the pandemic context as a response to the behavioral immune system. Sociosexual desire differs from sexual desire in that the former is targeted explicitly at potential mates to whom no committed romantic relationship exists [43],Penke and Asendorfp, 2008). Consequently, sociosexual desire can be interpreted as the motivational predisposition to allocate mating effort in short-term mating tactics (Penke and Asendorfp, 2008), and it is related to the diversity of mating partners. In this sense, we found an effect of the pandemic context but only in single individuals. That is, single men in prestudy 1 showed more sexual fantasies compared to the panstudy online sample but not to the panstudy face-to-face sample. Additionally, we established that single men in pre-pandemic study 2 had more fantasies than those who participated in the panstudy online but less than in panstudy face-to-face sample. These results, linked to the panstudy face-to-face sample, are inconsistent with the predictions. We can speculate that the experimental design of a panstudy face-to-face could affect those results, i.e., in this study, men were part of mixed-sex groups. Accordingly, the presence of women can bias the responses, increasing the perception of the frequency of sexual fantasies as a post-lockdown effect in men, which is coherent with the notion of sociosexual desire as the motivational predisposition to allocate mating effort in short-term mating tactics (Penke and Asendorfp, 2008). However, this problem was not present in our reduced (panstudy online) sample. We found an effect of the pandemic context but again only in single individuals, with men in prestudy study 1 reporting the most significant number of fantasies compared to individuals with high and low-risk perception. In addition, single men showed greater sociosexual desire in prestudy 2 compared to those who were at high risk of contagion but not with those who were at low risk. These results are more in accordance with our predictions since despite the variability across studies in the frequency of sexual fantasies, men reported a higher number of them in the previous period compared to the pandemic period, especially in those individuals with the perception of high risk of infection. Previous studies reported a decrease in libido that may be associated with a reduction in general sexual desire [7, 13] and may explain our results related to the decrease in sociosexual desire,unfortunately, we have not collected information about general sexual desire in our sample to test whether the reduction in desire was specific to sociosexual desire or was more general as previous studies suggest.

Regarding our fourth prediction, we expected reduced sociosexual behavior in single individuals during the pandemic compared with a non-pandemic period. However, we found that individuals before the pandemic period reported a higher number of last-year sexual partners regardless of their relationship status. This result is aligned with previous evidence that suggests a reduction in sexual activity, especially in single individuals [22],Hille, et al., 2021; [48]. In addition, when we analyzed risk perception, we found an effect of the context since individuals reported a more significant number of sexual partners before the pandemic, but only compared to subjects with higher risk perception. In addition, those with a low perception of contagion risk reported a more significant number of partners than the high-risk group. These findings show that the effect of the behavioral immune system over sexual behavior seems independent of relationship status and significantly affects men that perceive a high risk of contagion. In addition, and as occurred with sexual desire, we probably captured a post-lockdown effect with men who perceived a low risk of contagion. This effect has been previously described for a specific group of the population (see not heterosexual men in [16, 29, 42]), which we have not controlled in our sample. Another explanation could be raised considering the quarantine system adopted in Chile during the pandemic. In this sense, sociosexual behavior represents the behavioral expression of sociosexual attitudes given the opportunities and constraints present in the local environment [21]. The government of Chile established a system of dynamic quarantines between different geographic areas. These divisions were based on a plan decreed by the Ministry of Health that kept a large part of the country in total confinement for extended periods (between 90 and 172 days). It is possible that after the peak of lockdown, i.e,. During reduced restrictions conditions, the mating opportunities were increased, as has been observed in the Australian [16] and British [29] populations. This could explain why there are no differences in the sexual behavior of individuals with a low perception of risk during a pandemic.

In this study, we show that sociosexual desire and behavior are expressed in lower levels during the pandemic period compared to a period before the pandemic, however, the STMO were similar across periods. Previous studies reported a decrease in libido and sexual activities in single individuals during the pandemic [7, 19, 22, 48]. However, data in these studies was collected only during the pandemic or before lockdowns and isolation and therefore lacked data on sociosexual attitudes prior to the pandemic to compare with [30], which could reflect a bias in the results. In this sense, our study is the first that compares variables related to sociosexuality in two periods, i.e. previous non-pandemic and pandemic periods. Our results reflect that differences observed at the behavioral level could be more related to external sources, such as confinement measures, rather than to the activation of the behavioral immune system in terms of short-term sociosexuality. However, the fact that the sociosexual behavior were only decreased in those subsample of individuals with a high perception of contagion risk jointly with the evidence that sociosexual desire seems to be expressed in lower levels during the pandemic period point toward that the activation of the behavioral immune system could contribute to the decrease of sociosexual behavior beyond the effects due to confinement measures.

This study only contemplated the participation of men as a criterion because the baseline of previous studies only contemplated this study population. One of the premises of the behavioral immune system is that it is found universally in human beings [39]. In this sense, this psychological mechanism would have evolved as a way of saving resources for the biological immune system to detect the presence of pathogens [37]. That is why this work did not discriminate according to different characteristics of participants, such as ethnicity, educational level, or sexual orientation. We used the same criteria for the studies that served to construct the baseline. However in future studies, these differences must be incorporated in the analyses as control variables. In addition, for the case of the presence of mental and physical disorders, as exclusions criteria.

This study has several limitations. One of the limitations is that we did not include other psychological variables, such as personality characteristics, risk or germ aversion, which could also explain sociosexual behavior in contexts of risk of contagion by pathogens. We also did not measure stress or mental health symptoms associated with confinement that cause mental health problems [5]. We did not include other aspects that could affect sociosexuality, such as deaths in families or whether our participants were infected or not. Situations of loss and uncertainty, such as what was experienced in the pandemic, could affect people's sociosexuality. It would be worth exploring whether this had an impact on future studies. In addition, the effect of vaccines on sociosexual attitudes and behavior could be investigated. However, at the time of data collection from this study, only a portion of Chile's population was vaccinated primarily with the Sinovac vaccine, which was shown on the market with moderate efficiency. According to the predictions of the behavioral immune system [36], it would be plausible to think that vaccines could increase sociosexuality in the short term in men since there would be a protection derived from their use. We also used different sampling methods (face-to-face) and online, due to the restrictive conditions imposed by the pandemic period, however, most of the studies mentioned in this work used a similar strategy. In addition, we have not applied the COVID-19 survey to the face-to-face group, which is a limitation since this data was only present for the online sample.

In conclusion, this study provides partial support for the activation of the behavioral immune system. We found differences in long-term orientation, desire, and sociosexual behavior between pandemic and non-pandemic periods, particularly for single individuals. While these results could be explained alternatively by the lockdown and other isolation measures implemented during a pandemic, results regarding perceptions of high or low risk of contagion provide addition support for the notion of the activation of the behavioral immune system. This is the first study that explores all dimensions of sociosexuality (attitudes, desires, and behavior) and compares them between two periods in different individuals of the same country. This result will help enrich the debate about the activation and scope of the behavioral immune system in men.

## Supplementary Information

Supplementary Material 1.

## Data Availability

Data for this study are available at Open Science Framework OSF: https://osf.io/86qw3/?view_only = bddc01c07cfb4bb4a8a35f1c3bf6bd68. Also, all instruments were used to this study are available in supplementary materials.
