# Supplementary material for: The role of the behavioral immune system in the expression of short and long-term orientation in young Chilean men during the COVID-19 pandemic
Source: BMC Public Health. 2025 Feb 7;25:501. doi: 10.1186/s12889-025-21755-y (PMC11803946; doi:10.1186/s12889-025-21755-y)
Supplement: Supplementary file 1 — Supplementary Material 1. [file 12889_2025_21755_MOESM1_ESM.docx]

**Sociodemographic Information**

Below you will find a series of general questions that are relevant to the research you are participating in. These questions are not intended to identify you personally in any way.

Age (in years): _________

Please mark with an X the option that corresponds to your personal case:

Gender:

Male: ______

Female: ____

Other: _____

Not answer: ___

Current relationship status (mark with an X the option that corresponds to your personal case):

Married: _______

Cohabiting:______

Engaged: ______

Dating: ______

Single: _______

If you are in a relationship, how long have you been with your partner? (indicate years and/or months): ____________

Do you have children?

No: ______

Yes: _______ How many: _______

Do you have children with your current partner?

No: ______

Yes: _______ How many: _______

Are you pregnant?

No: ______

Yes: _______ How many weeks: _______

Sexual orientation? (mark with an X the option that corresponds to your personal case):

Exclusively Heterosexual______

Primarily Heterosexual_____

Exclusively Homosexual ______

Primarily Homosexual_____

Bisexual_______

Have you been vaccinated?

Yes ___

No _____ If you don’t, why? ___________________

**COVID-19 Risk Perception: Probability and Severity**

How likely do you think it is that you will get infected with COVID-19?

Extremely unlikely [*][*][*][*][*][*][*] Extremely likely

How susceptible do you think you are to getting infected with COVID-19?

Not at all susceptible [*][*][*][*][*][*][*] Very susceptible

How severe would it be for you to get infected with COVID-19 (how severe do you think your symptoms would be)?

Not severe at all [*][*][*][*][*][*][*] Very severe
